# A new chapter of Meiyu story: misty rains stopped and extremes amplified

**DOI:** 10.1093/nsr/nwae259

**Published:** 2024-08-07

**Authors:** Yihui Ding

**Affiliations:** National Climate Center, China Meteorological Administration, China

Climate conditions are crucially shaping the evolution of human civilization, because a distinctive climate creates diverse modes of production, lifestyles, and cultural behavior [1]. The traditional Meiyu was one such climate phenomenon influencing oriental civilization for thousands of years (Fig. 1a). The traditional Meiyu was characterized by non-extreme conditions with misty features of gentle and successive rain, muggy and high humidity environments [2]. However, it has been rare to see misty Meiyu in past recent decade. Just as every coin has two sides, the weakening of traditional Meiyu is also accompanied by extreme rainfall or severe heatwave-drought conditions that are always located on the two opposites of a single variable (e.g. precipitation or apparent temperature). For example, a super-strong Meiyu triggered devastating floods in eastern China and Japan in 2020; conversely, a long-lasting and blistering heatwave and drought covered the entire Yangtze River basin and the Japanese islands in 2022.

**Figure 1.**
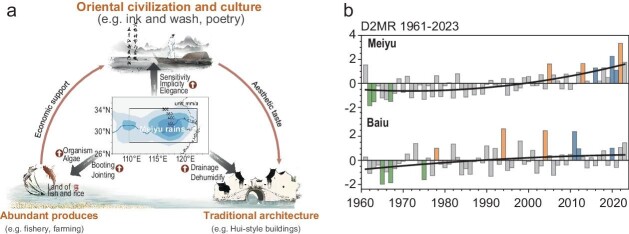
(a) Conceptual pathway of how the traditional Meiyu moulded the agroecosystem, culture, and architecture in the Yangtze River basin. The rounded arrows indicate enhancement. (b) Variation in D2MR (bars) averaged in the Meiyu and Baiu region and their trends during 1961–2023 (black curves). Green, orange and blue bars represent the extremely traditional Meiyu-Baiu, hot-dry and heavy-rain years. Adapted with permission from Ref. [4].

Previous studies mainly focused on increases in one side of extreme Meiyu [3]; however, these findings are unable to be generalized to understand the traditional misty rain and associated connotations. To-date, there is a lack of quantitative description of misty rain and thus no consensus on whether and to what extent the changes in the traditional Meiyu can be attributed to global warming.

Yin *et al.* [4] make important contributions to fill the gap in understanding between extreme and misty Meiyu and illustrates their relationships with anthropogenic activities. A degree of deviation to misty rains (D2MR) was constructed to innovatively solve problems of traditional Meiyu changes. The outstanding performance of D2MR comes from involving rich connotations of misty rains. The long-term increase of D2MR showed that the magic Asian Meiyu has almost completely lost its traditional and intrinsic misty-rains character both in the Yangtze River basin and Japanese islands (Fig. 1b). This suspension of traditional Meiyu-Baiu during 1961–2023 is mainly attributable to global warming as induced by anthropogenic activities. In the future, the Meiyu are projected to keep away from traditional regional and month-seasonal average rain amounts, replaced by amplified extreme rainstorms and drought. Destructive rainstorms and droughts are measurably and rapidly taking the place of the mild and maternal rain in East Asia; however, these sudden and destructive extremes may not allow ecosystems and human beings enough time to adapt. Therefore, it is of great concern to reveal that the East Asian Meiyu was no longer ‘mould rains’ and entered a new normal with amplified extremes [4].

As a distinct and far-reaching meteorological phenomenon, Meiyu has attracted worldwide research and a mass of relevant literatures have been published [2]. Creative and innovative work like that by Yin *et al*. [4] significantly broadens and advances Meiyu studies. In addition to the long-term trend, the variation of Meiyu also showed intraseasonal, interannual and decadal time-scale variabilities [2]. Differently, they were mainly modulated by western Pacific subtropical high, North Atlantic Oscillation, El Niño–Southern Oscillation, Pacific Decadal Oscillation and so on. Although real challenges exist, to improve multi-scale predictions of Meiyu would be highly beneficial for disaster prevention and risk management [2,5].

## References

[1] Carleton TA, Hsiang SM. Science 2016; 353: aad9837. 10.1126/science.aad983 27609899

[2] Ding Y, Liang P, Liu Y et al. JGR Atmospheres 2020; 125: e2019JD031496. 10.1029/2019JD031496

[3] Moon S, Utsumi N, Jeong J-H et al. Sci Adv 2023; 9: eadh419. 10.1126/sciadv.adh4195 PMC10672148 38000029

[4] Yin Z, Song X, Zhou B et al. Natl Sci Rev 2024; 11: nwae166. 10.1093/nsr/nwae166 38883297 PMC11173173

[5] Wang H, Dai Y, Yang S et al. Atmos Oceanic Sci Lett 2022; 15: 100115. 10.1016/j.aosl.2021.100115

